# Association of whale sharks (*Rhincodon typus*) with thermo-biological frontal systems of the eastern tropical Pacific

**DOI:** 10.1371/journal.pone.0182599

**Published:** 2017-08-30

**Authors:** John P. Ryan, Jonathan R. Green, Eduardo Espinoza, Alex R. Hearn

**Affiliations:** 1 Monterey Bay Aquarium Research Institute, Moss Landing, California, United States of America; 2 Charles Darwin Foundation, Puerto Ayora, Galápagos Islands, Ecuador; 3 Galápagos National Park Directorate, Puerto Ayora, Galápagos Islands, Ecuador; 4 Universidad San Francisco de Quito / Galápagos Science Center, Quito, Ecuador; Hawaii Pacific University, UNITED STATES

## Abstract

Satellite tracking of 27 whale sharks in the eastern tropical Pacific, examined in relation to environmental data, indicates preferential occupancy of thermo-biological frontal systems. In these systems, thermal gradients are caused by wind-forced circulation and mixing, and biological gradients are caused by associated nutrient enrichment and enhanced primary productivity. Two of the frontal systems result from upwelling, driven by divergence in the current systems along the equator and the west coast of South America; the third results from wind jet dynamics off Central America. All whale sharks were tagged near Darwin Island, Galápagos, within the equatorial Pacific upwelling system. Occupancy of frontal habitat is pronounced in synoptic patterns of shark locations in relation to serpentine, temporally varying thermal fronts across a zonal expanse > 4000 km. 80% of shark positions in northern equatorial upwelling habitat and 100% of positions in eastern boundary upwelling habitat were located within the upwelling front. Analysis of equatorial shark locations relative to thermal gradients reveals occupancy of a transition point in environmental stability. Equatorial subsurface tag data show residence in shallow, warm (>22°C) water 94% of the time. Surface zonal current speeds for all equatorial tracking explain only 16% of the variance in shark zonal movement speeds, indicating that passive drifting is not a primary determinant of movement patterns. Movement from equatorial to eastern boundary frontal zones occurred during boreal winter, when equatorial upwelling weakens seasonally. Off Peru sharks tracked upwelling frontal positions within ~100–350 km from the coast. Off Central America, the largest tagged shark (12.8 m TL) occupied an oceanic front along the periphery of the Panama wind jet. Seasonal movement from waning equatorial upwelling to productive eastern boundary habitat is consistent with underlying trophic dynamics. Persistent shallow residence in thermo-biological frontal zones suggests the role of physical-biological interactions that concentrate food resources.

## Introduction

### Environmental setting

**Fig 1:**
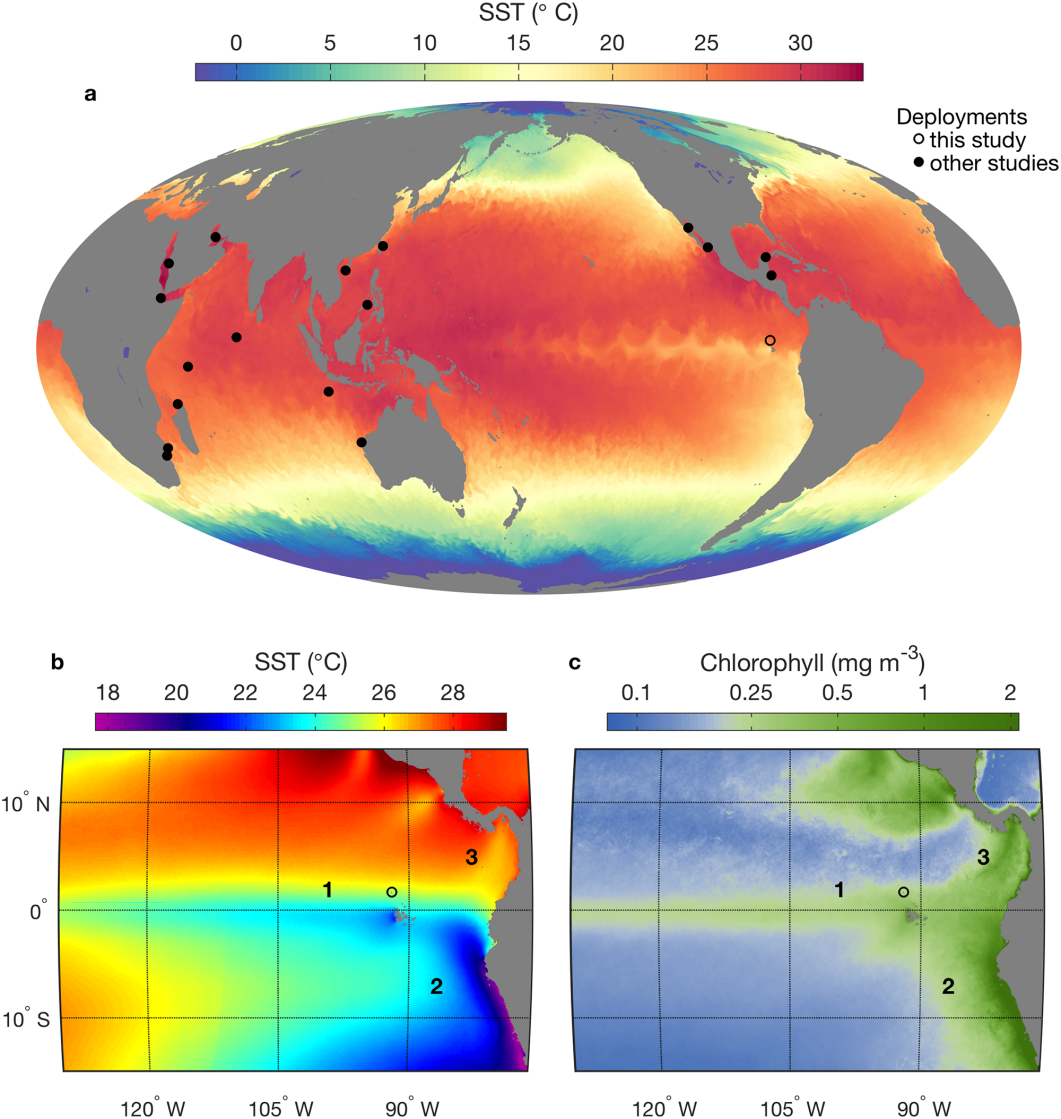
Global and regional context for satellite tracking studies of whale sharks. (a) Synoptic global sea surface temperature (SST) data represent the dynamic nature and large scale of the equatorial Pacific upwelling system, where whale sharks were tagged for this study. This NOAA 5-day analysis, centered on 15 October 2016, is from the time of year during which tracked whale sharks occupied equatorial habitat. The tag deployment site is near Darwin’s Arch (1.673°N, 91.989°W, open circle). Other sites from published studies where satellite tags have been deployed on whale sharks are shown (filled circles). (b,c) Long-term mean SST and surface chlorophyll-*a* concentrations derived from satellite remote sensing data. Numbers 1–3 identify thermo-biological frontal zones examined in relation to tracking data from whale sharks.

Within the eastern tropical Pacific is one of the most prominent structures in the global marine environment—the equatorial Pacific upwelling system (Fig 1a). Spanning nearly one quarter of the equatorial circumference [1], this upwelling system generates the highest average air-to-sea heat fluxes [2] and one quarter of global new primary production [3]. The dynamic nature of the equatorial Pacific is pronounced in synoptic patterns of sea surface temperature (SST). Wavelike structure in SST along the equatorial upwelling (Fig 1a) is due to tropical instability waves / vortex trains [4,5]. These dynamics determine patterns of poleward advection of cold water, nutrients, and enhanced phytoplankton biomass [6–9], as well as accumulation of plankton in frontal convergence zones through physical-biological interactions [4,10–12]. The whale shark tag deployment site for this study is located in the equatorial Pacific ecosystem, at the northern extent of the Galápagos Islands (Fig 1a).

The instantaneous structure of upwelling and mixing dynamics (Fig 1a) is erased by long-term averaging, revealing instead the structure and magnitude of time-integrated consequences (Fig 1b and 1c). The influence of processes that disrupt tropical ocean thermal stratification is evident as relatively cool SST (Fig 1b), and enhanced productivity resulting from the associated nutrient flux into the euphotic zone is evident as relatively high chlorophyll concentration (Fig 1c). The area immediately west of the Galápagos Islands exhibits the strongest open-ocean mean anomalies in the equatorial Pacific (Fig 1b and 1c), and this area can host the coldest open-ocean waters along the global equator (Fig 1a). Biological island mass effects in this area are attributed to enhanced nutrient supply to the euphotic zone, which arises from interactions between island forms and circulation of the ocean and atmosphere [13–16]. Adjacent to the physical and biological signatures of the equatorial Pacific upwelling system are those of a coastal upwelling system south of the equator (Fig 1), the Humboldt Current System (HCS). Among the four major eastern boundary upwelling systems of the Pacific and Atlantic, the HCS is unique in its influence reaching so close to the equator (Fig 1), in its strong forcing by the El Niño—Southern Oscillation (ENSO) phenomenon, and in its order-of-magnitude higher fish production [17].

The major open ocean and eastern boundary upwelling systems of the eastern tropical Pacific generate the strongest patterns in the long-term mean, and their associated frontal zones are prominent (labels 1 and 2 in Fig 1b and 1c). Darwin’s Arch, the whale shark tagging location for this study, is within the northern equatorial upwelling frontal zone (label 1 in Fig 1b and 1c). Additionally, a third type of frontal zone is evident. Physical consequences (oceanic eddy activity, upwelling, vertical mixing, cooling) and biological consequences (enhanced productivity and chlorophyll concentrations) result from wind jets that flow through mountain gaps in Central America and extend hundreds of kilometers over the Pacific margin [5,16,18–20]. The frontal zone created by the southernmost Central American wind jet system is off Panama (label 3 in Fig 1b and 1c).

The present study is the first examination of oceanographic dynamics underlying whale shark habitat occupancy in the eastern tropical Pacific, and in any major open ocean or eastern boundary upwelling system (Fig 1a). The goal of this study is to evaluate whether, within the vast tropical Pacific ecosystem, whale sharks exhibit preferential habitat occupancy in relation to the three types of productivity enhancing ecosystems and their associated thermo-biological frontal systems (labels 1–3 in Fig 1b and 1c).

### Satellite tracking of whale sharks in the eastern Pacific

Among approaches to studying the behavioral ecology of whale sharks (*Rhincodon typus*) is application of animal tracking technologies that reveal patterns of range, movement, and inhabitation (geographic, depth, hydrographic). Published studies of whale shark satellite tracking in the eastern Pacific have involved tag deployments in only two regions: the Gulf of California (GOC) and the Galápagos (Fig 1a). Tracking of 15 mostly immature whale sharks tagged in the GOC during 1994–1996 showed that most remained in the Gulf, however four left the Gulf and ranged widely in the North Pacific [21]. Eight tag deployments in GOC during 2008–2012 showed that the 3 juveniles remained in the Gulf, and the 5 adults moved out into the North Pacific [22]. In this later study, 4 of the 5 adults were female and apparently pregnant, leading the authors to suggest that parturition may occur in offshore waters [22].

**Fig 2:**
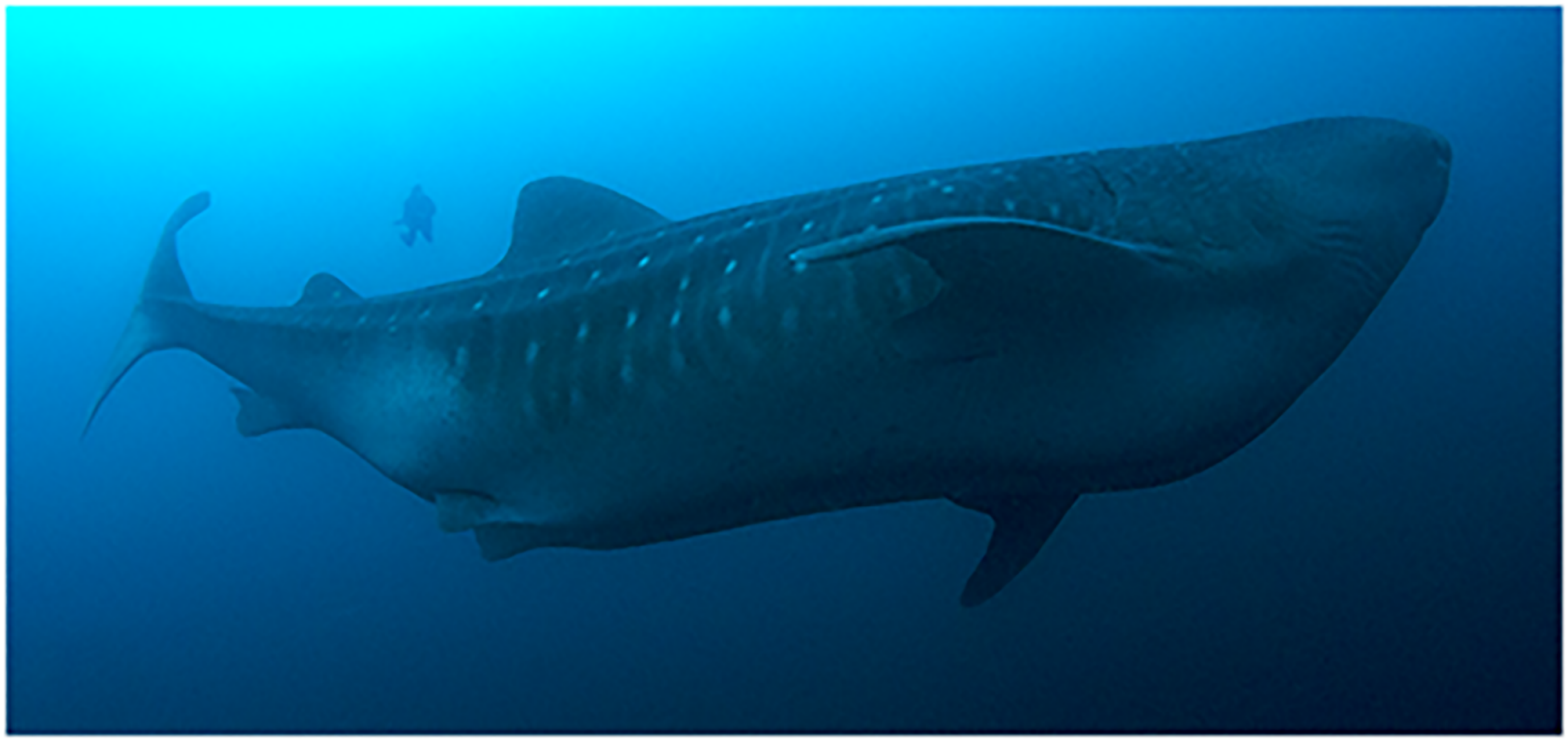
Photograph of a whale shark, *Rhincodon typus*, near Darwin’s Arch, Galápagos. This 12.5 m TL apparently pregnant female was tagged on 7 September 2012 (WS-31 in [23]).

Deployment of satellite tags in the Galápagos Islands in 2011–2012 provided tracking data for 27 whale sharks [23]. Presence of whale sharks near Darwin Island, at the northern reach of the archipelago, has been observed mostly between July and November, a temporal pattern shared with hammerhead (*Sphyrna lewini*) and Galápagos (*Carcharhinus galapagensis*) sharks [24,25]. During these months waters are coldest, chlorophyll concentrations reach their highest levels, and equatorial currents and fronts are relatively strong [26]. Whale sharks evidently remained in the tagging area for only days before moving into the open equatorial Pacific, and those returning from the open Pacific to eastern boundary habitat again exhibited short-term residence around Darwin [24]. Photo identification and total length measurements of 82 whale sharks off Darwin Island during 2011–2013 showed that 92% were adult females, all but one of which exhibited a distended abdomen (e.g. Fig 2) suggestive of pregnancy [23–25]. Although apparently pregnant females have been observed in the northeastern Pacific [22,27] and southeastern Atlantic [28], the highly skewed demographic observed in Galápagos is exceptional.

## Materials and methods

### Ethics statement

The work undertaken for the preparation of this manuscript was approved by the University of California Davis Institutional Animal Care and Use Committee (IACUC) Protocol # 16022 and authorized by the Galápagos National Park Directorate research permit PC-37-11.

### Whale shark tag deployment and data

The methods used to tag and track the whale sharks whose habitat occupancy patterns are examined in this study, as well as descriptions of each shark, have been published elsewhere [23,29] but are briefly summarized here. Whale sharks were tagged off Darwin Island, Galápagos, at a location known as Darwin’s Arch (1.673° N, 91.989° W). Scuba diving was used to attach towed satellite tags (SPOT-5 and SPLASH tags, Wildlife Computers, WA, USA; and SeaTags, Desert Star Systems, CA, USA). All tags were tethered on 1.2–2 m steel cable anchored to the dorsal musculature of the sharks with titanium barbs. Divers recorded gender and estimated length using photogrammetry techniques [30].

In the context of the present study, a key point is that all satellite tags provided high-accuracy location data, with accuracies > 250 m to > 5 km depending on the number of successive transmissions received by the satellite [31,32]. High-accuracy position data are critical to relating the sharks to evolving features in a dynamic habitat. Distance between each successive point was calculated using Vincenty’s formula in the geosphere package in R [33,34]. An iterative forward / backward averaging filter [35] was applied to remove locations with large error [36,37]. For the resulting tracks, tag detachment was determined based on timing and quality of message criteria [29]. A single point for each day was selected based on quality (2 and 3 were preferred over A and B) and proximity to midday.

Subsurface time-at-temperature (TAT) data were provided by a subset of tags for the equatorial Pacific. Tags provided summaries for 12-hour periods, set to coincide with dawn and dusk in Galápagos (6 am and 6 pm respectively), although as sharks crossed multiple time zones, it was not possible to analyze the data from a diel perspective. The summaries provided the percentage of time spent in temperature bins; bin boundaries were 0, 5, 10, 15, 18, 20, 22, 24, 26, 28, 30, >30°C. Tag temperature resolution reported by the manufacturer (Wildlife Computers) is 0.05°C, and accuracy is ±0.1°C.

### Environmental data analysis

To examine time-varying habitat conditions in dynamic marine habitat relative to whale shark occupancy, optimizing spatial and temporal resolution of environmental data is essential. For SST observations this can be achieved by integrating strengths of infrared (superior spatial resolution) and microwave (superior temporal coverage) remote sensing. This study used daily SST from combined microwave / infrared data, obtained from Remote Sensing Systems [38]. Daily SST data were averaged to 3-day means to reduce noise while retaining sufficient temporal resolution to examine shark locations in relation to dynamic environmental features. To visually represent synoptic distributions of sharks in relation to meandering fronts across large regional expanses, 3-day segments of shark tracking data are shown in relation to the corresponding 3-day mean SST. All SST and position data matchups are available in a supplementary animation [39], and a representative subset is presented for each ecosystem.

To quantitatively examine shark positions in relation to frontal gradients in the equatorial upwelling system, meridional SST sections were extracted from the SST images at the longitude of and nearest in time to each shark position. Sections are presented for each shark to examine consistency. Transformation of the meridional spatial reference from latitude to distance relative to the shark was applied in all spatial analyses. This was required because the latitude of the front varies greatly (~ 5°). Thus, the spatial relationships in a latitude reference frame become confused for any plot showing multiple sections, and the actual structure encountered by the sharks becomes smeared in computing a mean profile or any statistical description. Translation of latitude to distance relative to the shark solves this problem.

Two aggregated analyses are presented. The first quantifies the spatial relationship between each shark position and the front, computed as percent distance between the equatorial and northern sides of the front. The equatorial side of the front is defined by the temperature minimum caused by equatorial upwelling. Maximum SST gradients extend from this temperature minimum northward, typically hundreds of kilometers, until a distinct break in the slope. This slope change defines the northern side of the front. This environmental structure and analysis method are illustrated with the results. The second aggregated analysis is a 2D histogram computed at a resolution of 0.5°C and 50 km. This probability density distribution of SST in a shark-centric reference frame complements the examination of individual profiles by shark and augments consideration of the ecological relationship. For the coastal upwelling system, a subset of tracking data supported a similar analysis, except that the sections were zonal and at the latitude of the sharks. However, the much smaller set of position data for the coastal system does not motivate the 2D histogram analysis.

To illustrate long-term mean SST (Fig 1b) monthly microwave / infrared data from 2002–2013 [40] were temporally averaged. Unlike combined infrared / microwave SST, ocean color remote sensing is severely limited by cloud cover in this region. The length of time required to derive a relatively complete sampling within the domain surrounding a shark far exceeds the duration of local occupancy by the shark, and it smears the environmental features encountered by the shark. Although ocean color data are too sparse to contribute to the emphasis of this study on relatively rapid changes in the distributions of sharks and environmental features, we use the strength of long-term ocean color observation to clearly define the thermo-biological frontal systems that whale sharks were observed to occupy (Fig 1c). Chlorophyll concentration data were 5-year mean (2012–2016) from the Visible Infrared Imaging Radiometer Suite (VIIRS, 750 m resolution), obtained from the NASA Ocean Biology Processing Group at Goddard Space Flight Center [41].

To evaluate the extent to which zonal shark movements in the equatorial Pacific were influenced by ocean currents, we produced a matchup data set based on the shark tracking data and Ocean Surface Current Analysis Real-time (OSCAR) data [42,43]. Because the equatorial current system is predominantly zonal (E-W), and the tracked whale sharks exhibited large-scale zonal movements, this analysis focused on zonal currents only. For each shark the speed of zonal movement was computed from daily position data, limited to position pairs having temporal separation of 3 days or less. This included most position data but avoided high uncertainty from estimating shark movement speed across large gaps in tracking. Zonal distance was computed between longitudes of each position pair using their mean latitude (distance function in MATLAB). Zonal surface current speed was then extracted at the OSCAR grid location nearest each position pair (OSCAR spatial and temporal resolutions are 1/3° and 5 days). The matchup data set was examined in two ways: (1) comparison of histograms, and (2) computation of the coefficient of determination (r^2^).

Although no in situ observations were collected across frontal gradients in the study region as part of this project, such observations are periodically collected as part of the Tropical Atmosphere Ocean (TAO) program. The TAO data archive [44] was searched for the entire tracking period to identify ship-based observations near in space and time to habitat occupancy by any of the tracked whale sharks. This produced one matchup in which a whale shark moved westward across a north-to-south ship survey along 110°W. Data from this cruise (KA-11-04) examined here include CTD observations of hydrography and Acoustic Doppler Current Profiler (ADCP) observations of current velocity.

## Results

### Tag deployment overview

**Fig 3:**
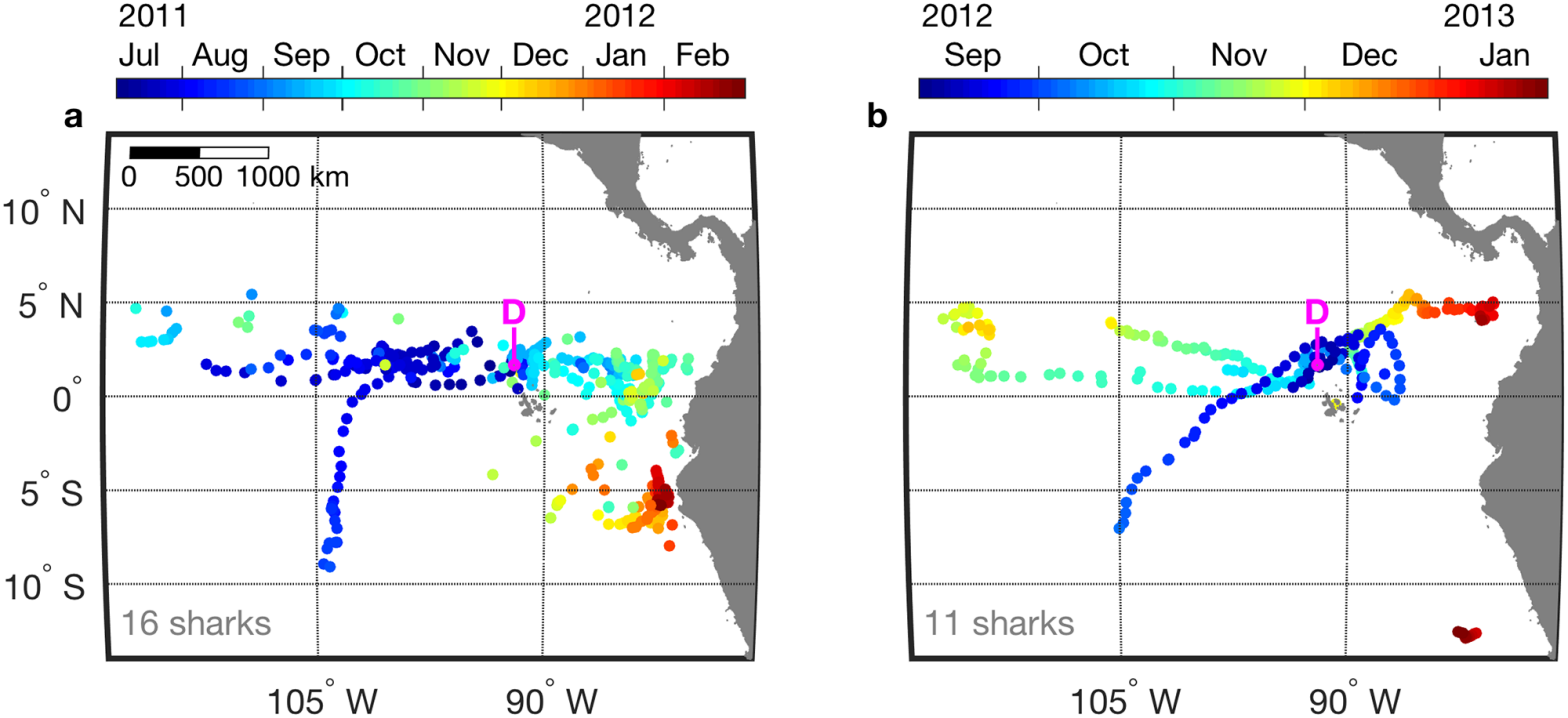
Whale shark tracking summary. Daily positions are colored according to time for each tracking period: (a) 8 July 2011 to 1 March 2012, and (b) 3 September 2012 to 25 January 2013. The number of sharks tracked is indicated in each panel. The location labeled ‘D’ is the tag deployment site, Darwin’s Arch (1.673°N, 91.989°W).

Data from 27 tags are examined in this study, 16 deployed during July—October 2011, and 11 deployed during September—October 2012 (Fig 3). The domain defined by shark position data spans 4060 km zonally and 2027 km meridionally. The first tracking period (Fig 3a) was 3 months longer than the second (Fig 3b), and it included 5 more tags, contributing to greater spatial and temporal coverage during the first tracking period. Earlier studies characterized the tracked population [23–25]. All tracked sharks were female except one, and a distended abdomen (Fig 2) suggestive of pregnancy was a prevalent observation. Estimated total lengths ranged from 4.0 to 12.8 m. Tracking durations ranged from 7 to 176 days (mean = 63 days), and the number of daily positions derived from each tag ranged from 2 to 134 (mean = 25). A seasonal pattern of residence described for the first tracking period [23] is represented in Fig 3a, with the population occupying equatorial habitat west of Galápagos during July—October 2011, and equatorial and ocean margin habitat east of Galápagos during November 2011 –February 2012. During the second tracking period, occupation of eastern margin habitat again occurred during the latest period of tracking (Fig 3b; January 2013). This involved two sharks tracked east of 85°W, widely separated in latitude (Fig 3b; ~5°N and 12°S). During each tracking period one sub-adult female shark moved far south of the equator near 105°W (Fig 3, different sharks in the two tracking periods).

### Association of whale sharks with the northern equatorial upwelling front

**Fig 4:**
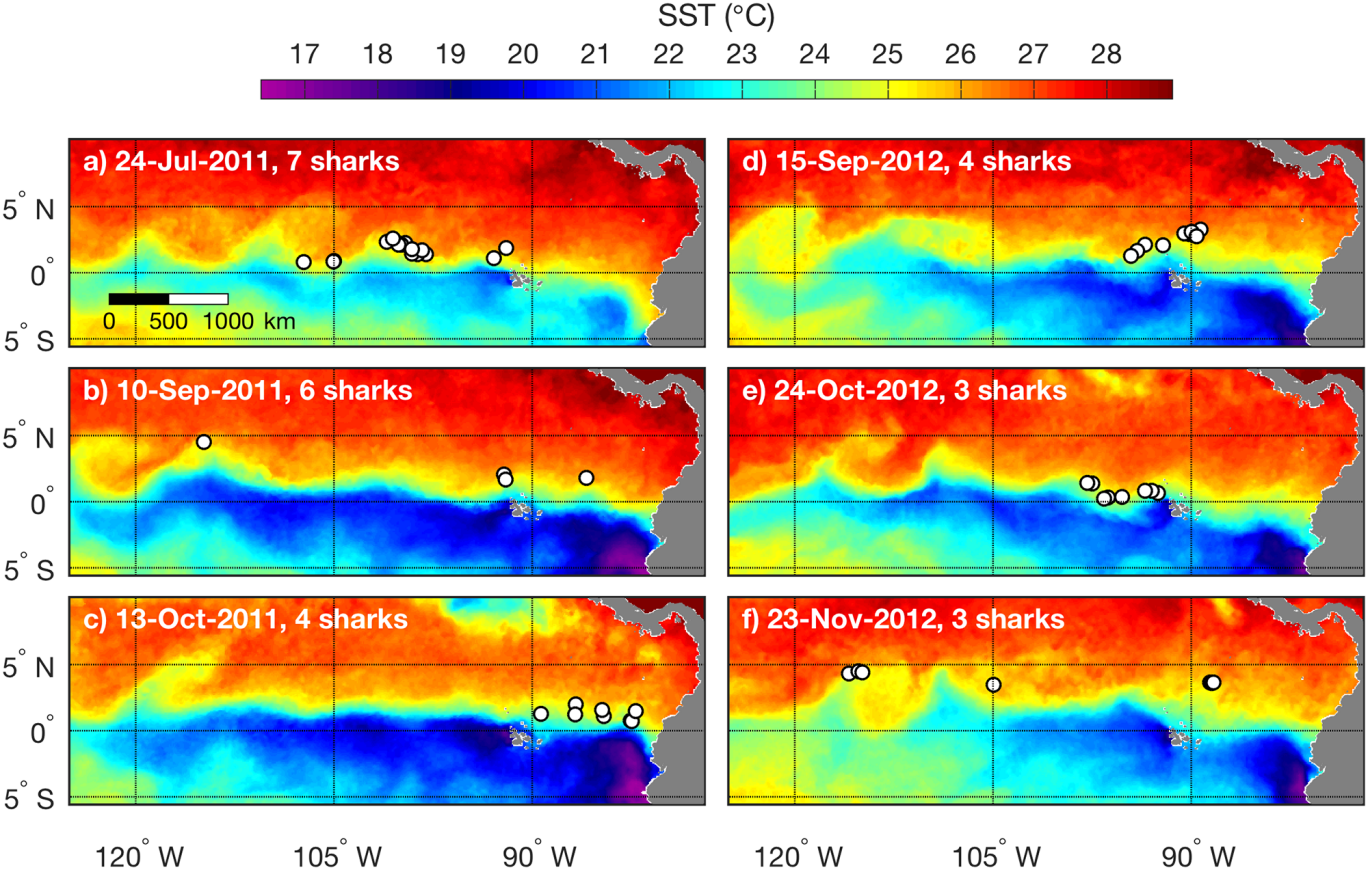
Association of whale sharks with the northern equatorial upwelling front. Synoptic examples are from the first (a-c) and second (d-f) tracking periods (Fig 3). Dates on images are the mid-point of 3-day mean SST, and corresponding shark positions during each 3-day period are overlaid (white circles). The number of sharks represented in each image is noted.

The majority of tracking data are from northern equatorial habitat (Fig 3). In this region whale sharks were consistently located in water near 25°C, between the cold water from equatorial upwelling and warmer water to the north. Representative examples of this frontal association from each tracking period are shown in Fig 4, and an animation showing all tracking illustrates its consistency throughout the periods of equatorial habitat occupancy [39]. Because the latitude of the front varies widely due to variability in upwelling, and to wave and eddy phenomena along the equator, the frontal association spanned a meridional distance exceeding 500 km. For example, frontal zone occupancy near the equator is evident in Fig 4a,4c and 4e, and frontal zone occupancy near 5°N is evident in Fig 4b and 4f. At any time, frontal habitat occupancy could span a large zonal and meridional expanse. For example, simultaneous frontal occupancy by 6 sharks spanned 3,000 km zonally and 300 km meridionally on 10 September 2011 (Fig 4b). Also apparent in synoptic distributions is the close association between serpentine distortions of the front, caused by instability waves / vortices, and the whale shark distributions. The 3-day summary centered on 24 July 2011 (Fig 4a) illustrates a cluster of four whale sharks centered near 100°W and aligned NW-SE with the front that had been tilted by vortex circulation. Similarly, the 3-day summary centered on 15 September 2011 (Fig 4d) illustrates a cluster of four whale sharks aligned with an oppositely tilted front (SW-NE).

**Fig 5:**
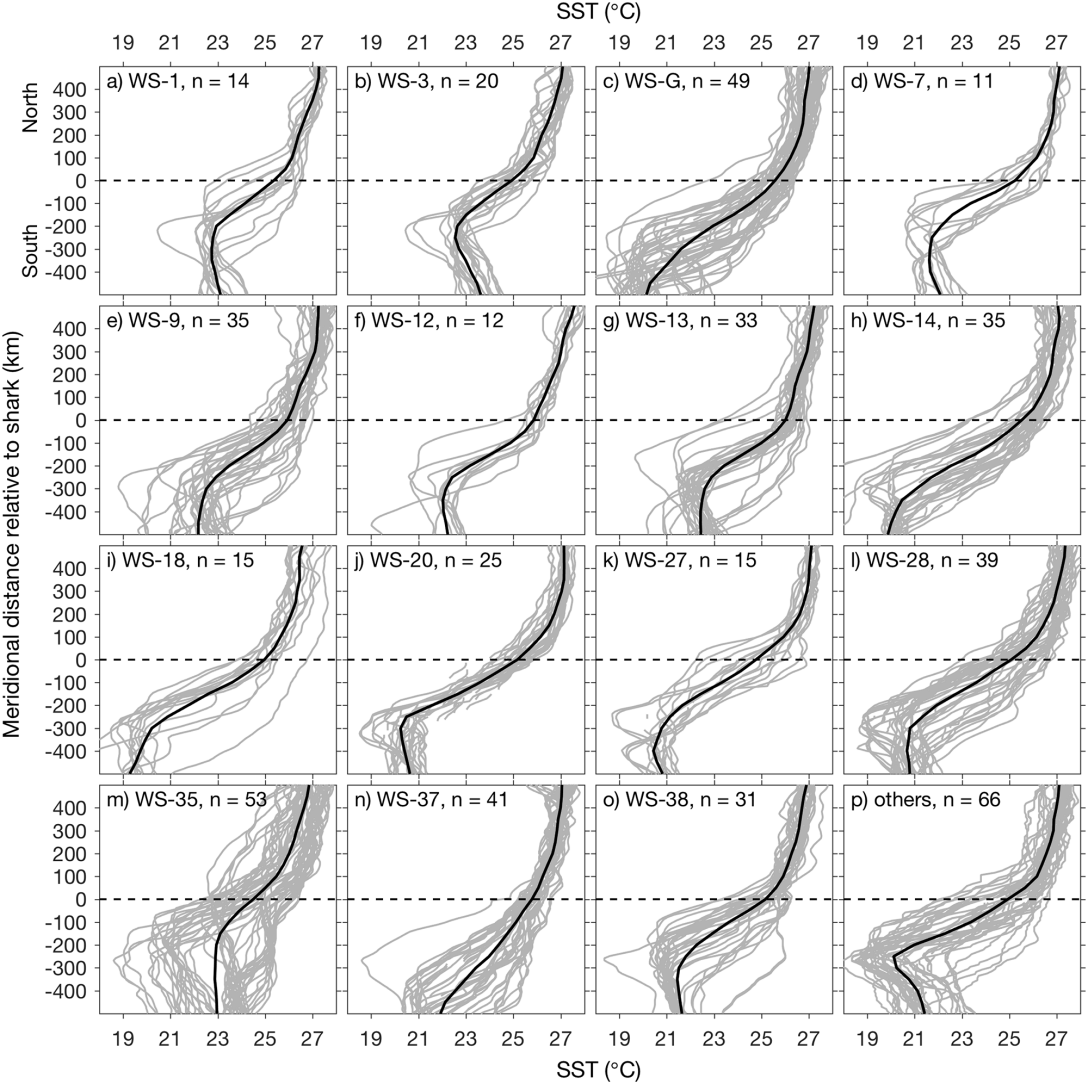
Association of whale sharks with the northern equatorial upwelling front. Meridional SST profiles (gray lines) are shown for each shark tag that provided > 10 positions (a-o) and separately for all shark tags that provided fewer positions (p). Means for each subset are overlaid (black lines). WS-G is “George” (c), the only male whale shark tracked.

SST gradients in northern equatorial habitat are primarily oriented zonally (Fig 4). Thus, meridional SST sections across shark positions can define the relationship between the sharks and the equatorial upwelling front. Because of the large latitude range of the frontal movement (Fig 4), examination of frontal structure relative to the sharks cannot effectively use latitude as a consistent spatial reference. A more effective reference frame is meridional distance relative to the shark location. SST sections for all northern equatorial tracking in this reference frame show predominant occupancy within the upwelling front (Fig 5).

The northern equatorial upwelling front extends from the temperature minimum near the equator, northward to where a large decrease in the meridional SST gradient occurs. For example, considering the SST profiles for WS-3 (Fig 5b), SST minima were ~ 250 km south of the shark positions, the steepest SST gradient extended from the temperature minima to ~ 100 km north of the shark positions, north of which there was a transition to a weaker thermal gradient (slope break). This general pattern of shark location (zero distance) within the strong SST gradient of the northern equatorial upwelling front was evident for all sharks (Fig 5). Some exceptional patterns are also apparent. For example, relatively warm water south of WS-35 (Fig 5m) was related to the circulation of a tropical instability wave vortex, which distorted the frontal boundary and transported warm water south of the shark (Fig 4f, westernmost positions). Exceptionally cold water extending far south of WS-G (Fig 5c) was related to influence of the cold plume emanating from the Peru upwelling, which merged with equatorial upwelling.

**Fig 6:**
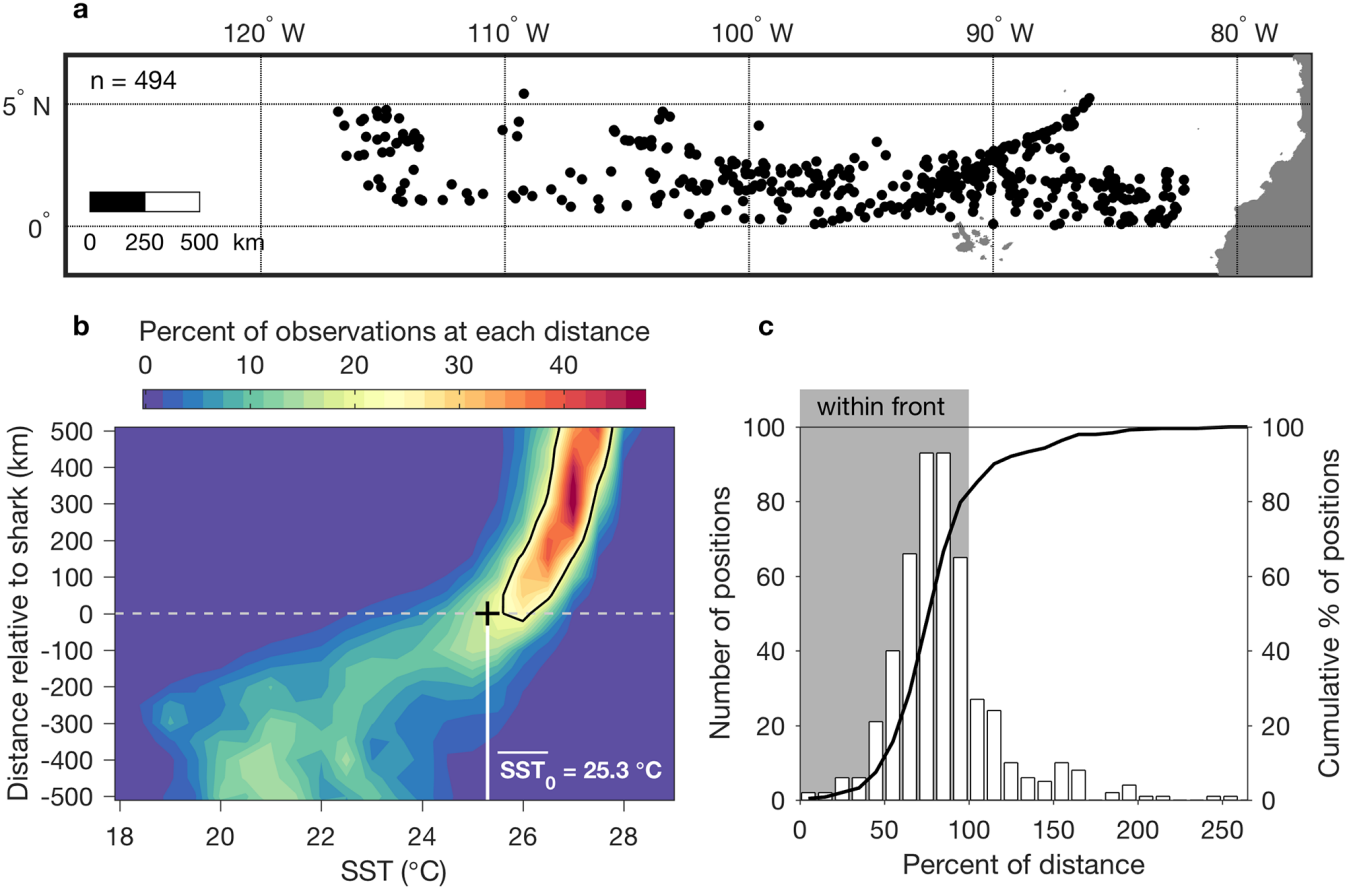
Statistical summary of northern equatorial frontal occupancy. Results are from both tracking periods. (a) The map shows the subset of daily position data examined (n = 494). (b) 2D histogram of meridional SST sections across all shark positions. The distance axis represents meridional distance relative to the shark, with zero at the shark position and positive distance north. Mean SST at all shark locations is noted and marked by the vertical white line. (c) Histogram of shark positions in relation to the upwelling front. Positions < 100% are in the front; positions > 100% are north of the front (see Materials and Methods).

The apparently strong relationship between whale sharks and the northern equatorial upwelling front (Fig 5) is examined in aggregate statistically (Fig 6). Shark positions included in these analyses spanned thousands of kilometers along the equator (Fig 6a). The 2D histogram (Fig 6b) is effectively a probability density distribution of meridional SST profiles. The percentage of observations within a distance—SST bin (resolved at 50 km and 0.5°C) is quantified at each distance from the shark. It represents the count at each distance—SST bin, divided by the sum of observations at that distance. This result indicates that the sharks tend to inhabit a transition point in environmental stability, near a mean temperature of 25.3°C (Fig 6b). The histogram of SST changes markedly at the location of the sharks. SST values north of the sharks fall within a relatively narrow temperature range, forming a ridge in the probability density distribution. In contrast, SST values south of the shark (toward equatorial upwelling) are distributed across a wider temperature range, representing influence of heat fluxes that originate with upwelling. Distance measurements show that 80% of all shark positions were within the upwelling front (Fig 6c). Further, the sharks tended to occupy the warmer half of the front, with 72% of all positions located between 50 and 100% of the distance between the equatorial temperature minimum and the northern side of the front. Including the region immediately adjacent to the northern side of the front, where plankton accumulation processes occur, the front and its outer periphery (0–120% distance) contain 90% of all shark positions (Fig 6c).

**Fig 7:**
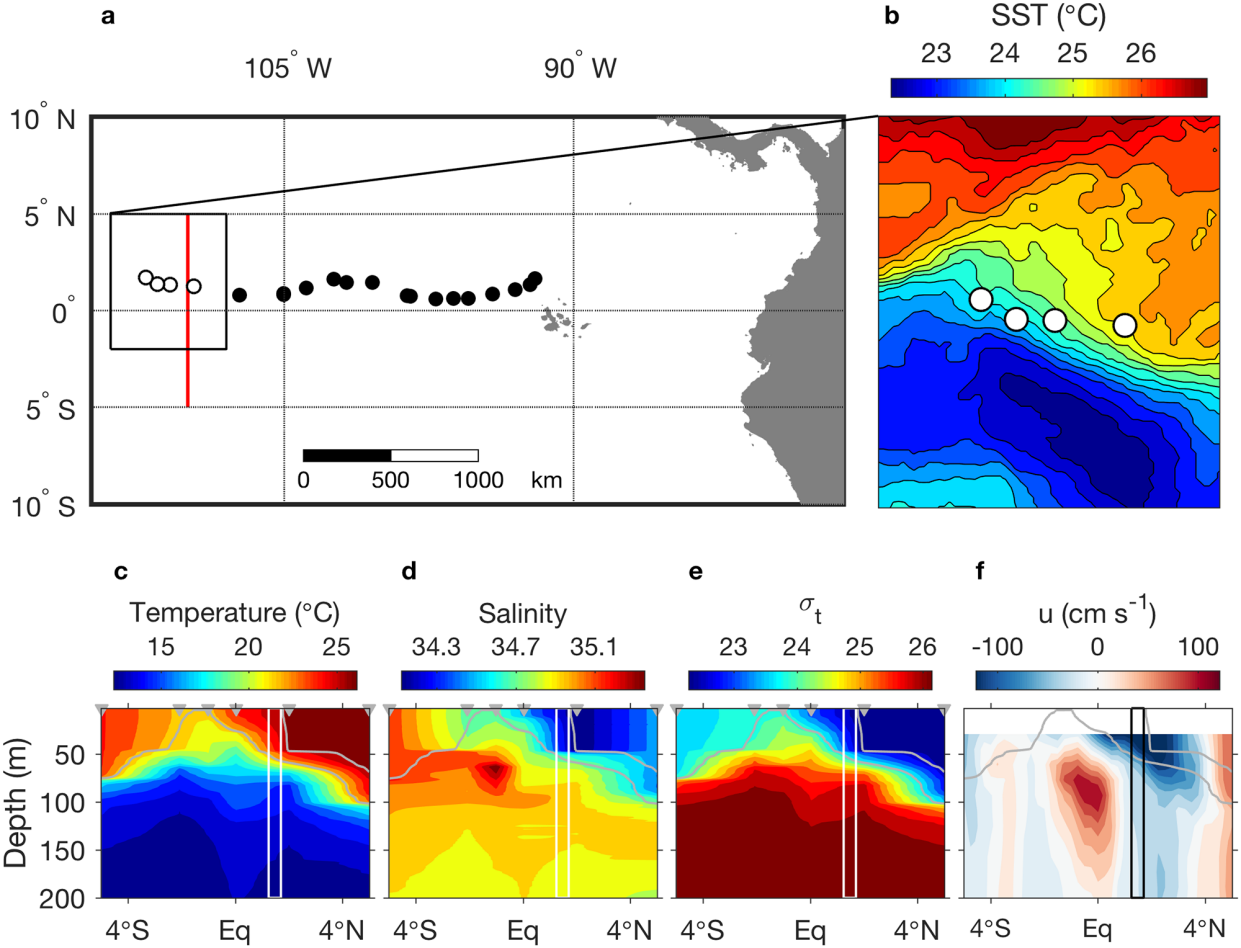
Surface and subsurface observations of whale shark habitat. Surface and water column observations across the northern equatorial upwelling front when occupied by a 4 m TL female whale shark (WS-3 in [23]). (a) Map shows track of WS-3 during westward movement. The final four positions (open circles) coincided with a ship survey along 110°W (red line). These four positions were acquired 26–29 July 2011 and are overlaid on SST for the 3-day period centered on 27 July 2011 in (b). The 5°N to equator portion of the full ship section (most relevant to the shark habitat) was occupied between 30 July and 2 August 2011. (c-f) Water column properties along the ship section; gray triangles mark the locations of the CTD profiles; sigma-t represents density; u is zonal velocity. The vertical lines mark the latitude range occupied by WS-3 between 26 and 29 July 2011. Gray contours mark the temperature range 22 to 26°C (Fig 8a).

Examination of in situ environmental data in relation to frontal occupancy by a whale shark was possible for one individual, an immature female (4 m TL; WS-3 in [23]). During July 2011, westward movement of this shark crossed the north-south transect of a ship survey along 110°W (Fig 7a). Surface and subsurface temperature data show residence along the warm side of the upwelling frontal zone (Fig 7b and 7c), consistent with the pattern in synoptic examples (Fig 4) and aggregated analyses (Figs 5 and 6). The vertical lines in Fig 7c–7f define the latitude range of the shark during its westward movement across the ship transect (westernmost four positions in Fig 7a). This latitude range was immediately south of the warmest / freshest / least dense water in the mixed layer (Fig 7c–7e), a transition point in environmental stability (Fig 6b).

**Fig 8:**
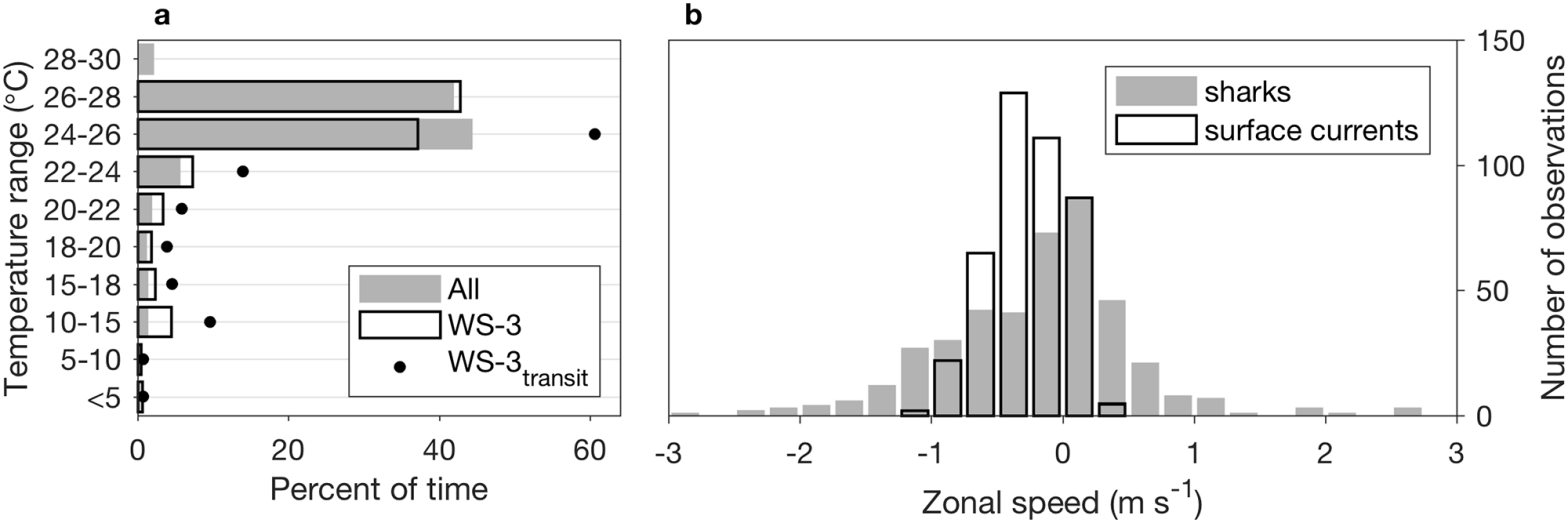
Temperature range occupation and zonal current influence in the equatorial region. (a) Subsurface temperature analysis is based on TAT data from 13 tags. Represented separately are WS-3 tag data and the subset of WS-3 tag data during which the shark transited across a ship survey (Fig 7a). (b) Histograms of zonal speeds of (1) shark movements and (2) surface currents at shark locations.

Subsurface temperature data from this tag, during the period of tracking highlighted in Fig 7a, show that the shark mostly occupied a narrow temperature range, spending 75% of the time between 22°C and 26°C (WS-3_transit_ in Fig 8a). This temperature range is shown in the ship temperature section (Fig 7c–7f, gray contours), emphasizing occupancy of near-surface habitat; the 22°C isotherm was shallower than 60 m within the latitude range of the shark. The temperature histograms for this subset period were similar to those for the entire tracking of WS-3, indicating persistent occupancy of warm (shallow) water (Fig 8a). Considering the entire track of WS-3, the shark occupied waters > 22°C 87% of the time. Further, the temperature histogram for the full WS-3 track is similar to that from all subsurface temperature data in the equatorial upwelling system (Fig 8a, All; 13 tags), which define occupancy in waters > 22°C 94% of the time. The coldest temperature recorded in this data set was below 5°C (Fig 8a). Four sharks occupied these coldest temperatures at different places and times. At the location where the ship intersected the track of WS-3 in late July 2011 (Fig 7a), temperatures of 5°C or lower were below 900 m.

In the area of the ship transect, WS-3 resided within the northern lobe of the South Equatorial Current (SEC), which had maximum westward velocities exceeding 1 m s^-1^ below 30 m (Fig 7f). The average speed of the shark along the full westward trajectory shown in Fig 7a was 1.4 m s^-1^. This example suggests influence of the SEC on westward movement of WS-3. This potential influence is examined in relation to surface zonal current estimates collocated with all equatorial shark positions (Fig 8b). Consistent with the influence of the northern lobe of the SEC, westward (negative) zonal currents were prevalent where the sharks were located (Fig 8b, surface currents). The histogram of the zonal speeds of shark movement was skewed toward negative (westward) also, consistent with influence of the SEC. However, the zonal speeds of the sharks spanned a much larger range, and 40% of them were directed eastward (opposite the SEC). Further, surface zonal current speeds explain only 16% of the variation in the zonal speeds of the sharks (r^2^ = 0.16 in matchup data set of n = 266). Thus, the strong association between sharks and the northern equatorial front was not simply a consequence of passive drifting in the SEC.

### Association of whale sharks with the eastern boundary upwelling system front

**Fig 9:**
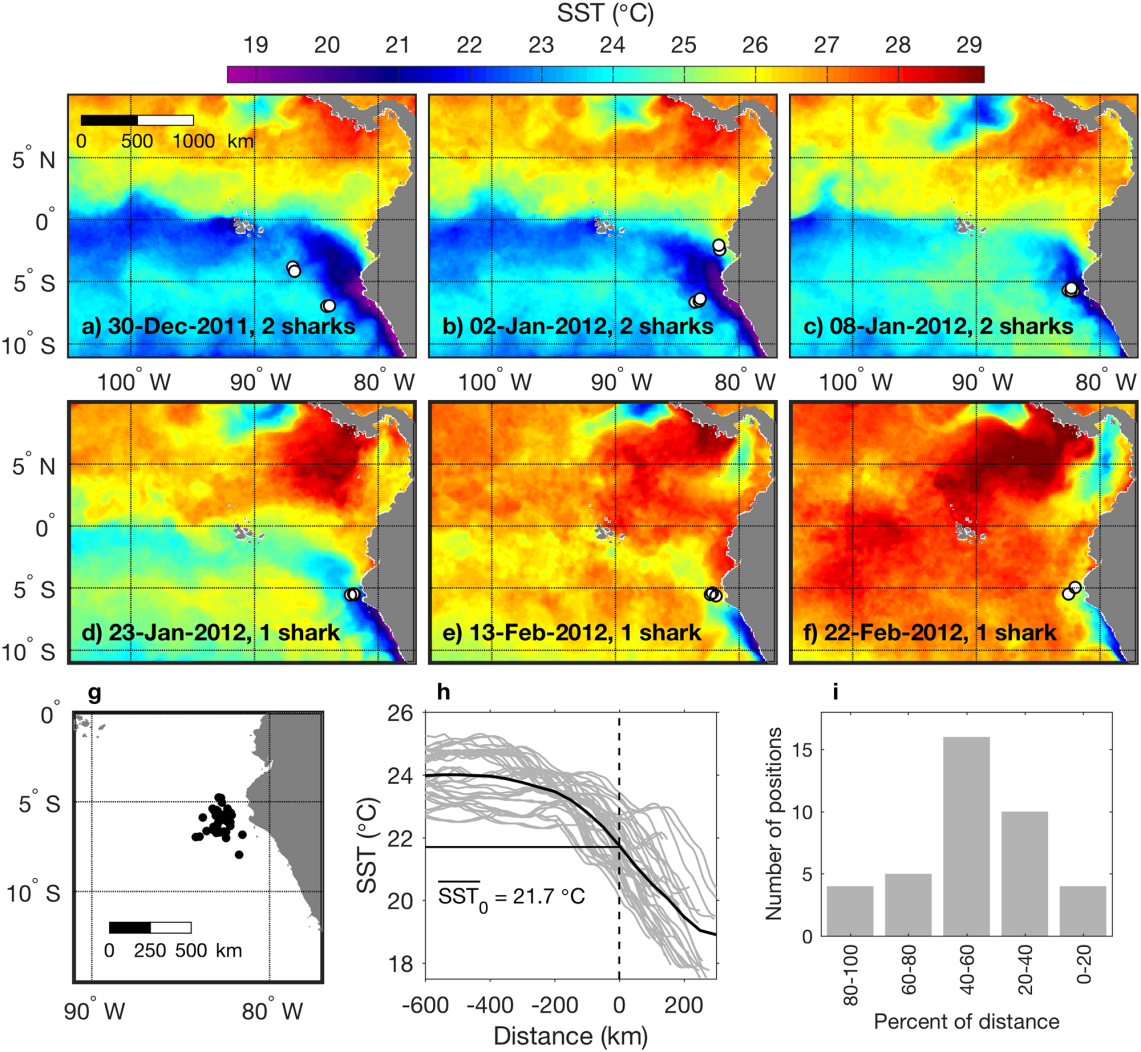
Association of whale sharks with the eastern boundary upwelling system front. (a-f) Synoptic examples of SST and shark position data. Data are from the first tracking period (Fig 3a). Dates on images are the mid-point of 3-day means, and corresponding shark positions during each 3-day period are overlaid (white circles). The number of sharks represented in each image is noted. (g) The map shows a subset of position data for which zonal sections could effectively resolve SST gradients across eastern margin habitat. (h) Zonal SST sections across each shark position (gray lines) and the mean (black line). The distance axis represents zonal distance relative to the shark, with zero at the shark position and positive distance east. Mean SST at the shark locations is noted and marked by the horizontal line. (i) Location of shark positions relative to the front; percent distance is between the temperature minimum (caused by coastal upwelling) and the SST gradient slope break (e.g. near 200 km west of the shark positions in the mean profile in (h)).

During the first tracking period (Fig 3a) four sharks moved to the eastern boundary near 5°S off Peru during late December 2011 through late February 2012. Positions between the two upwelling ecosystems comprised only 8% of the tracking data for these sharks. All position / SST images can be viewed in the supplementary animation [39], which illustrates persistent occupancy of the periphery of upwelling plumes in the HCS. A representative subset of synoptic distributions illustrates how this pattern of occupancy occurred from hundreds of kilometers offshore (Fig 9a) to very close to the coast (Fig 9c–9f). Occupancy by multiple sharks also occurred simultaneously on both southern and northern (offshore and inshore) boundaries of the coastal upwelling plume that separates from the coast near 5°S (Fig 9b). During this period upwelling weakened, and the region warmed (Fig 9a–9f). By late January 2012 only one shark was being tracked, and it remained near 5°S along the northernmost periphery of the narrow coastal upwelling plume (Fig 9d–9f).

A subset of the tracking data in the HCS (Fig 9g) allowed examination of habitat occupancy with respect to the zonal thermal gradient (Fig 9h). Positions represent four whale sharks and distances between ~100 and 350 km from the coast. Of the 39 positions, 31 are from an 11.4 m female (WS-14 in [23]); 6 of the remaining 8 positions are from a 10 m female (WS-22). Seasonal variation (Fig 9a–9f) spread mean temperature among SST sections, yet in terms of SST gradients the sharks were always located within the upwelling front (Fig 9i, all between 0 and 100% of the distance between the temperature minimum from coastal upwelling and the slope break at the offshore periphery of the front). In the HCS, which exhibits lower average temperatures than the equatorial upwelling system (Fig 1b), mean SST at the shark locations was cooler, 21.7°C (Fig 9h), compared to the equatorial mean of 25.3°C (Fig 6b). In contrast to the equatorial region where sharks tended to occupy the warmer half of the front (Fig 6c, 50–100% of distance), maximum occupancy in the coastal region was in the center of the front (Fig 9i, 40–60% of distance).

### Association of a whale shark with a wind-jet system oceanic front

**Fig 10:**
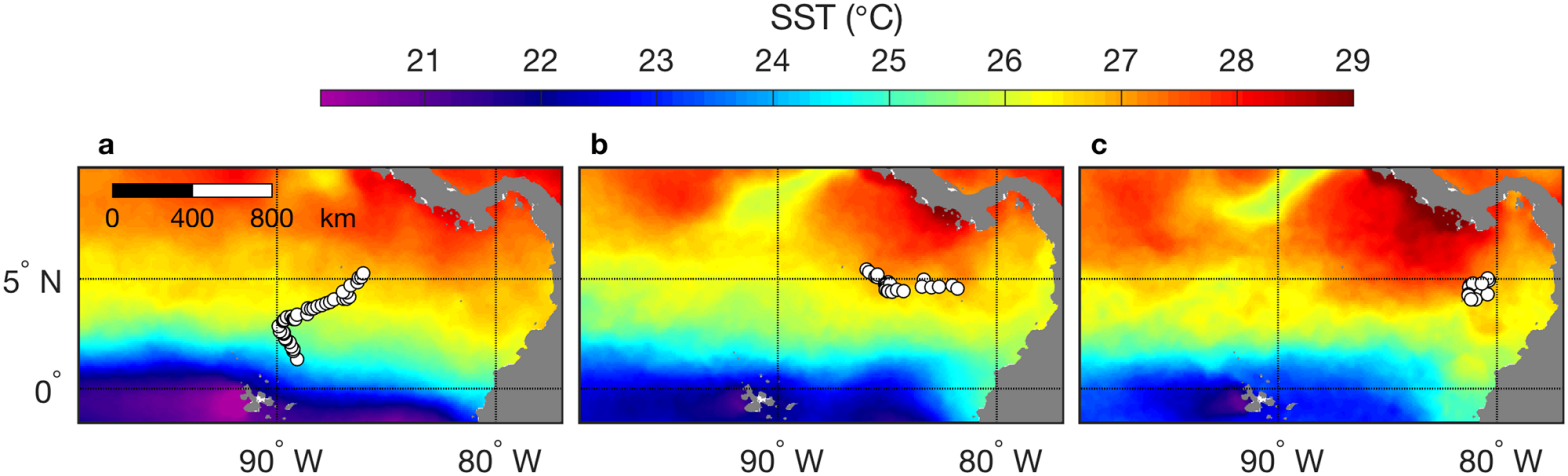
Occupancy of a wind-jet system oceanic front. Tracking data are from the largest whale shark in this study (WS-37 in [23]; 12.8 m TL) during (a) 31 October to 9 December 2012, (b) 10 December 2012 to 3 January 2013, and (c) 4 to 18 January 2013. SST images represent the average for the corresponding tracking periods.

One whale shark showed movement and habitat occupancy different from all others, reaching the farthest northeastern extent of tracking during January 2013 (Fig 3b). Following tagging in mid-October 2012, this 12.8 m female (WS-37) exhibited three phases of movement. During the first phase the shark moved steadily north (first NW, then NE), away from equatorial upwelling, until 9 December 2012 (Fig 10a). During the second phase the shark moved eastward toward the Gulf of Panama during 10 December 2012 to 3 January 2013 (Fig 10b). During the third phase the shark remained within a relatively small region from 4 to 18 January 2013 (Fig 10c), after which tracking ceased. Indications of Panama jet influence on the oceanic habitat are evident in mean SST during the period when the shark remained near 5°N, 80°W (Fig 10c). Specifically, the area east of the shark was cooler, and this relatively cool feature extended northward to the coast of Panama, narrowing toward the coast. This same pattern is evident in long-term mean SST (Fig 1b), it can be much more pronounced in synoptic observations (e.g. Fig 9f), and it is consistent with the typical pattern resulting from wind jets that develop in this region [5,20]. Associated with this wind jet is a strong biological front along which chlorophyll concentrations are elevated in the mean (label 3 in Fig 1c).

## Discussion

The whale sharks tracked in this study were associated with three ecosystems of the eastern tropical Pacific: (1) the open ocean equatorial upwelling system, (2) the eastern boundary upwelling system of the South Pacific, the Humboldt Current System, and (3) the Central American wind jet system nearest the equator. These three ecosystems represent the primary physical processes that break down nutrient-limiting stratification that would otherwise prevail beneath tropical insolation. Upwelling and wind-driven mixing, which interact with large-scale oceanic internal dynamics driven by Kelvin and Rossby waves, enhance primary productivity [6,7]. The heat fluxes and biological enrichment of these environments also create thermo-biological fronts along the margins of their influence. It is these frontal zones that appear to be preferred habitat for whale sharks.

Following tag deployment in Galápagos, the sharks could have ranged anywhere in the eastern tropical Pacific. Instead, they remained predominantly within frontal habitat. In the northern equatorial region 80% of positions were within the upwelling front, and in the HCS 100% of positions were within the upwelling front. This high proportion of habitat occupancy in upwelling fronts, disproportionate relative to the area of the eastern tropical Pacific that is comprised of upwelling frontal habitat, indicates preferential occupancy of a specific habitat type. Sharks that occupied both upwelling systems had 92% of their tracking data within the upwelling habitats and only 8% between them, further indicating preferential occupancy. In the equatorial Pacific the association with frontal habitat closely followed serpentine distortions of the equatorial front, with simultaneous frontal occupancy by multiple sharks ranging over hundreds of km meridionally and thousands of km zonally. In the HCS the association with frontal habitat closely tracked the scale and spatial pattern of the coastal upwelling plume. The association with frontal habitat across both upwelling ecosystems was thus strong in a spatial as well as a temporal context. A relatively small amount of tracking data was returned from the third type of thermo-biological front, off Central America. Yet this data also indicates the importance of frontal habitat, as the largest tracked shark lingered in this habitat type for at least two weeks following months of steady movement to the eastern margin.

Large marine ectothermic planktivores face a major challenge to find prey in tropical oceans where plankton availability is variable in space and time [45,46]. To make feeding energetically viable, they require high-density prey patches [46]. Whale sharks form feeding aggregations at a number of coastal locations around the world, which are timed to coincide with high local productivity, such as the annual mass spawning of red land crabs at Christmas Island [47,48], sergestid shrimp off Tanzania [46], or copepods off Baja California, Mexico [49]. However, whale shark populations at these locations are mostly composed of juvenile males. In the open ocean, frontal zones may provide the most efficient means of obtaining food, especially for large females, which tend not to associate with seasonal coastal productivity.

Association of other fish species with frontal zones has been observed. For example, the planktivorous filter feeding basking shark (*Cetorhinus maximus*) has been observed to selectively forage in the densest plankton patches within frontal zones of the NE Atlantic [50,51]. GPS tracking of an ocean sunfish, *Mola mola*, along an 800 km migration path in the California Current System revealed persistent occupancy of upwelling frontal habitat, whether strong fronts near the coast or weak fronts further offshore [52]. Synthetic aperture radar imagery showed slicks indicating convergence within these fronts, which can accumulate the gelatinous zooplankton prey of the sunfish, and high concentrations of salps were observed in frontal zones near the tracked *M*. *mola*. An individual short ocean sunfish, *Mola ramsayi*, tagged in the western region of the Galápagos Islands, moved along the northern equatorial upwelling front similarly to the whale sharks in this study [53]. Other fish species extending across trophic levels—anchovy, sardine, jack mackerel, albacore—have been found to aggregate in frontal zones [54,55]. In the eastern tropical Pacific, association with upwelling fronts is also evident in distributions of seabirds. A 10-year study based on 45 meridional sections showed that planktivorous seabirds were more concentrated in the northern equatorial front than in adjacent waters, and their population densities increased with frontal intensity [56].

Upwelled waters are cold and impoverished of plankton, thus making them disadvantageous habitat in terms of thermoregulation and foraging efficiency. Beyond the domain of upwelling influence, where waters remain strongly stratified and relatively plankton-poor, habitat may be disadvantageous primarily because food resources are relatively sparse. Thus, frontal zones between these endmember waters are somewhat expectedly a balancing point where food may be relatively abundant and energy requirements for thermoregulation may be relatively low. Within this zone and along the mean horizontal transport pathway away from the upwelling, plankton communities grow and mature. The most efficient places for foraging by planktivores such as whale sharks may be locations where physical-biological interactions concentrate mature plankton communities.

In the equatorial upwelling system, long-term mean chlorophyll concentrations show a maximum along the equatorial divergence. This is evident in satellite remote sensing data (Fig 1c) and confirmed by in situ observations, which also show that zooplankton biomass is usually highest several degrees north and south of the equator [57]. The spatial offset between phytoplankton and zooplankton abundance maxima reflects the product of poleward meridional flow [58] and temporal lag of zooplankton community response to phytoplankton primary productivity. This offset also informs understanding of habitat occupancy by planktivores that feed mostly on zooplankton, such as whale sharks. Mature zooplankton communities will develop in the same region where physical fronts are created by poleward transport of upwelled water, toward the outer reaches of upwelling influence. Physical-biological interactions may also concentrate trophic resources. For example, positively buoyant diatoms accumulate in convergence zones of tropical instability waves / vortex trains in the eastern tropical Pacific, toward the outer reaches of upwelling influence, creating sharp biological features that are visible from space [4,10,11], and buoyant foraminifera also exhibit abundance maxima within frontal zones in this region [12]. Viewing the SST gradients of this ecosystem from the perspective of the moving population, this study finds that most positions were within the northern half of the front and the immediately adjacent warm habitat to the north (89% between 50% and 120% of frontal distance). The probability density distribution of meridional SST surrounding all whale sharks tracked in northern equatorial Pacific habitat illustrates that this preferred habitat is also at a transition point in environmental stability, consistent with the outer reaches of upwelling influence.

High-resolution simulations of the physics of tropical instability vortices in the equatorial Pacific show that a large portion of the vortex periphery can exhibit convergent flow and frontogenesis [59]. The largest area of intense convergence develops along the western—northwestern boundary (the leading boundary in terms of vortex propagation). This is the region of the vortices that create extremely dense accumulations of diatoms through the interaction of convergent flow and diatom buoyancy [10]. Yet smaller patches of intense convergence develop along the northeast flank of vortices, and a large area of the southern periphery can be strongly convergent (Fig 12 of [59]). Whale sharks of this study were observed along the equatorial front where the frontal orientation was zonal as well as strongly tilted / distorted along the leading and trailing periphery of vortices. Thus, the physics of these vortices, an essential part of the ecosystem, are central to the highly dynamic shaping of whale shark foraging habitat.

Whale sharks in Galápagos tend to be far more abundant at the northern reaches, around Darwin and Wolf Islands, than elsewhere in the archipelago [23–25]. These northernmost islands are more closely coupled with the northern equatorial front than are the islands closer to the equator. This major frontal system extends great distances west and east of Darwin and may represent a primary and favorable migration corridor. The whale sharks do not stay at Darwin for long, also consistent with the overarching importance of a migratory corridor. In the Atlantic, mostly adult whale sharks are regular visitors to the Azores, which lie close to a thermal boundary [60], similar to northern Galápagos. It is unclear how these sharks navigate over long distances, but comparison of tracks with passive diffusion models off Ningaloo (Australia) suggested that their movements and speeds were not explained by geostrophic currents, leading researchers to hypothesize that thermoreception of large water temperature gradients associated with fronts and eddies may be used for navigation [61]. Examination of zonal surface currents and zonal shark movements in our study also emphasizes shark movement independent of major currents amid strong thermal gradients. Alternatively, sharks may orient to geomagnetic gradients while making long distance movements, a phenomenon termed “topotaxis” [62]. Although the exact sensory mechanisms involved in this have not yet been established, it remains a plausible hypothesis. Whether discernible in environmental conditions or their gradients, this dynamic corridor may be navigated using the highly evolved sensing capabilities of whale sharks.

Similar to patterns in the equatorial upwelling system, zooplankton abundances in the Humboldt Current System exhibit a maximum downstream of the upwelling source waters, offshore in deep water, i.e. off the continental shelf [63]. Observations show the importance of physical processes and associated water column structure across trophic levels in the northern HCS, including the region where we observed whale sharks to inhabit [64]. The structures are caused by processes across a range of scales, including internal waves, eddies and fronts. Mean biomass of zooplankton and fish was estimated to be ~150% and ~950% higher, respectively, within structures than in adjacent unstructured regions [64]. Stronger aggregation for fish was attributed to magnification of physically induced spatial structuring by behavior, and seabird foraging in a small-scale structured trophic hotspot was observed with seabird tracking and high-resolution in situ acoustic data.

Small schooling planktivorous fish, which exhibit abundances an order of magnitude higher in the northern HCS than in the other major eastern boundary upwelling systems [17], can constitute a trophic resource for whale sharks [65]. Thus ecological structures created by boundary current dynamics in the northern HCS may concentrate multiple trophic levels upon which whale sharks may forage. The frontal zones inhabited by whale sharks ranged from very nearshore to hundreds of kilometers offshore, which would presumably influence exposure to different schooling fish populations. Sardines and anchovies use the upwelling habitat differently, with sardines preferring oceanic waters and the upwelling frontal zone, and anchovies preferring colder waters closer to the upwelling source [66,67]. A key aspect of habitat structuring drawn out by this tracking of whale sharks was concurrent occupancy of frontal zones both offshore and inshore of upwelling plumes that separate from the coast in the northern HCS, near 5°S. This area of the coastal upwelling system also exhibited the densest occupancy by sharks, and it was the location where the longest tracking of a whale shark in the HCS remained as upwelling waned. These observations may indicate locally enhanced persistence and areal coverage of the physical-biological interactions that can create trophic hotspots.

Movement of whale sharks from equatorial to eastern boundary habitat when the equatorial upwelling seasonally weakens is consistent with an underlying trophic basis, perhaps moving to more favorable foraging habitat. The two destinations observed for whale sharks migrating toward the eastern boundary, the northern HCS off Peru and the wind jet system off Panama, exhibit seasonal variations in chlorophyll [68] that are also consistent with a trophic basis for the movement patterns. Specifically, movement of whale sharks to the HCS off Peru occurred during December 2011, and habitation in this region persisted at least until March 2012. Maximum chlorophyll concentrations in this region are observed during January—March [68], and whale sharks are known to occur off the coast of northern Peru during this period [69]. Movement of the largest tagged whale shark (WS-37) to the Gulf of Panama occurred during December 2012, and habitation in this region persisted at least into January 2013. Maximum chlorophyll concentrations in this region are observed during January—April [68]. The association of whale sharks with enhanced productivity in eastern boundary habitat is thus evident in a temporal as well as spatial (Fig 1c) context.

Additional ecological considerations are introduced by the fact that most (92%) of the whale sharks photo-identified and measured off Darwin Island during 2011–2013 were adult females, all but one of which exhibited signs of potential pregnancy [25]. Tracking of whale sharks in the Gulf of California also involved apparently pregnant females, which moved out of the Gulf and into the open North Pacific, leading the authors to suggest that parturition may occur in offshore waters [22]. If the parturition occurs in frontal habitats of the eastern tropical Pacific, the same attributes of this habitat that make it favorable for adults (energetics of thermoregulation and foraging efficiency) may apply to neonates and young. If young whale sharks feed close to the surface, we might expect that more neonates would be recorded in fisheries. Only around 20 neonate and young (<1.5 m) whale sharks have been reported to date across the globe [65,70,71]. Two of these were found in the stomachs of pelagic predatory fish—blue marlin [72] and a blue shark [73], whereas the others were all reported in open ocean habitat [73–75]. Four small whale sharks were caught in pelagic purse seines in the Eastern Pacific, a 93 cm female near Clipperton (10°N, 110°W), a 55cm female further west (10°N, 132°W), a 63 cm female 400 km off the coast of Costa Rica, and a 62 cm male caught 250 km off the coast of Guatemala [74].

The lack of evidence for connectivity between Galápagos and the Gulf of California is of particular interest because of known occupancy by apparently pregnant whale sharks [21,22] and relatively close proximity to Galápagos (Fig 1). Reproductive and trophic aspects of whale shark ecology may involve a migratory connection between these places. Although tracking studies have not yet revealed whale shark migration between these locations, this may be due simply to the limitations of tracking duration imposed by the technology of tag attachment and retention. Establishing longer tracking records in the eastern Pacific is an important goal for advancing understanding of whale shark ecology. In addition to the questions about geographic connectivity, population responses to the tremendous environmental variability imposed by ENSO motivates more intensive and enduring tracking. The tracking data collected during July 2011 –January 2013 coincided with La Niña conditions and may thus represent a specific mode of habitat use in the region.

## Conclusions

The first tracking study of whale sharks in the eastern tropical Pacific has yielded valuable insights into the demographics and ecology of this species within some of the most productive habitats in the global ocean. As noted in a recent review article [65], “The world’s largest shark … remains an enigma 183 years after first being described.” Although much has been learned, important questions remain in understanding the behavioral ecology of the largest extant fish species. As is true of other shark species threatened by exploitation, informing protection of this endangered species requires greater understanding of its ecology. By integrating advances in technology—tags with accurate long-duration tracking and environmental sensing capabilities, and multidisciplinary environmental sensing from satellite borne sensors and autonomous platforms that can follow tagged populations—we will advance the knowledge required to inform protection.
