# Correction: Association of whale sharks (*Rhincodon typus*) with thermo-biological frontal systems of the eastern tropical Pacific

**DOI:** 10.1371/journal.pone.0196443

**Published:** 2018-04-23

**Authors:** John P. Ryan, Jonathan R. Green, Eduardo Espinoza, Alex R. Hearn

There is an error in affiliation 2 for author Jonathan R. Green. Affiliation 2 should be: Fundación Megafauna Marina Ecuador, Whale Shark Investigation, Quito, Pichincha, Ecuador.
